# 5-[(3,5-Dichloro­anilino)meth­yl]-*N*-(3,5-dichloro­phen­yl)-6-methyl-2-phenyl­pyrimidin-4-amine

**DOI:** 10.1107/S160053681204665X

**Published:** 2012-11-24

**Authors:** Jerzy Cieplik, Janusz Pluta, Iwona Bryndal, Tadeusz Lis

**Affiliations:** a Department of Organic Chemistry, Wrocław Medical University, 9 Grodzka St, 50-137 Wrocław, Poland; b Department of Applied Pharmacy, Wrocław Medical Uniwersity, 38 Szewska St, 50-137 Wrocław, Poland; c Department of Bioorganic Chemistry, Faculty of Engineering and Economics, Wrocław University of Economics, 118/120 Komandorska St, 53-345 Wrocław, Poland; d Faculty of Chemistry, University of Wrocław, 14 Joliot-Curie St, 50-383 Wrocław, Poland

## Abstract

In the title compound, C_24_H_18_Cl_4_N_4_, the pyrimidine ring makes dihedral angles of 19.1 (2), 4.1 (2) and 67.5 (2)°, respectively, with phenyl and two benzene rings, and the mol­ecular conformation is stabilized by an intra­molecular N—H⋯N hydrogen bond closing a six-membered ring with an *S*(6) motif. In the crystal, a pair of inter­molecular N—H⋯N hydrogen bonds connect two mol­ecules, forming inversion dimers with *R*_2_^2^(12) motifs. C—H⋯π inter­actions links the dimers into a chain running along the *a-*axis direction. There are also π–π stacking inter­actions [centroid–centroid distance = 3.666 (4) Å] between the benzene rings of adjacent chains.

## Related literature

For the anti­bacterial activity of 6-methyl-2-phenyl-5-substituted pyrimidine derivatives, see: Cieplik *et al.* (2003 ▶, 2008 ▶). For related structures, see: Cieplik *et al.* (2006 ▶, 2012 ▶).
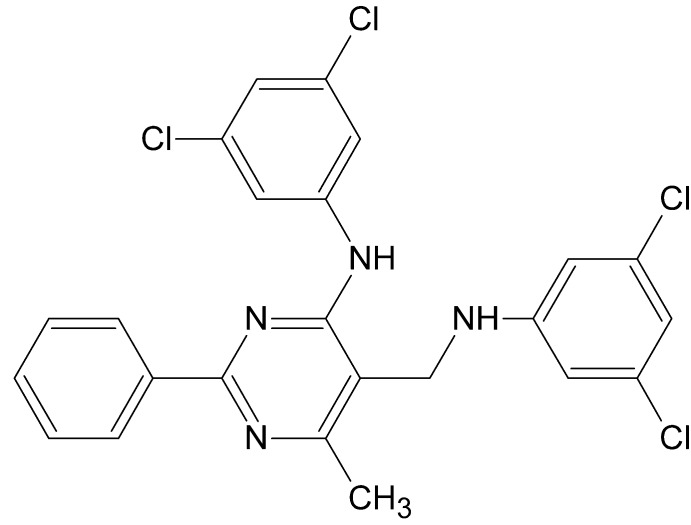


## Experimental

### 

#### Crystal data

C_24_H_18_Cl_4_N_4_
*M* *_r_* = 504.22
Triclinic, 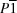

*a* = 8.267 (3) Å
*b* = 11.271 (4) Å
*c* = 12.788 (4) Å
α = 76.53 (5)°
β = 78.95 (5)°
γ = 81.42 (5)°
*V* = 1130.4 (7) Å^3^
*Z* = 2
Mo *K*α radiation
μ = 0.54 mm^−1^
*T* = 85 K
0.23 × 0.14 × 0.08 mm

#### Data collection

Oxford Xcalibur PX with Onyx CCD diffractometer
19215 measured reflections
8041 independent reflections
5082 reflections with *I* > 2σ(*I*)
*R*_int_ = 0.041

#### Refinement

*R*[*F* ^2^ > 2σ(*F* ^2^)] = 0.044
*wR*(*F* ^2^) = 0.100
*S* = 1.03
8041 reflections
296 parameters
H atoms treated by a mixture of independent and constrained refinement
Δρ_max_ = 0.75 e Å^−3^
Δρ_min_ = −0.41 e Å^−3^

### 

Data collection: *CrysAlis CCD* (Oxford Diffraction, 2007 ▶); cell refinement: *CrysAlis CCD*; data reduction: *CrysAlis RED* (Oxford Diffraction, 2007 ▶); program(s) used to solve structure: *SHELXS97* (Sheldrick, 2008 ▶); program(s) used to refine structure: *SHELXL97* (Sheldrick, 2008 ▶); molecular graphics: *XP* in *SHELXTL* (Sheldrick, 2008 ▶); software used to prepare material for publication: *SHELXL97*.

## Supplementary Material

Crystal structure: contains datablock(s) I, global. DOI: 10.1107/S160053681204665X/is5217sup1.cif

Structure factors: contains datablock(s) I. DOI: 10.1107/S160053681204665X/is5217Isup2.hkl

Supplementary material file. DOI: 10.1107/S160053681204665X/is5217Isup3.cml

Additional supplementary materials:  crystallographic information; 3D view; checkCIF report

## Figures and Tables

**Table 1:** Hydrogen-bond geometry (Å, °) *Cg*1 is the centroid of the C21–C26 ring.

*D*—H⋯*A*	*D*—H	H⋯*A*	*D*⋯*A*	*D*—H⋯*A*
N4—H4⋯N5	0.85 (2)	2.19 (2)	2.875 (3)	137.6 (17)
N5—H5⋯N1^i^	0.81 (2)	2.40 (2)	3.171 (3)	158.1 (19)
C57—H572⋯*Cg*1^ii^	0.99	2.65	3.62 (2)	166

Symmetry codes: (i) 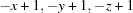; (ii) 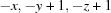.

**Fig. 1.**
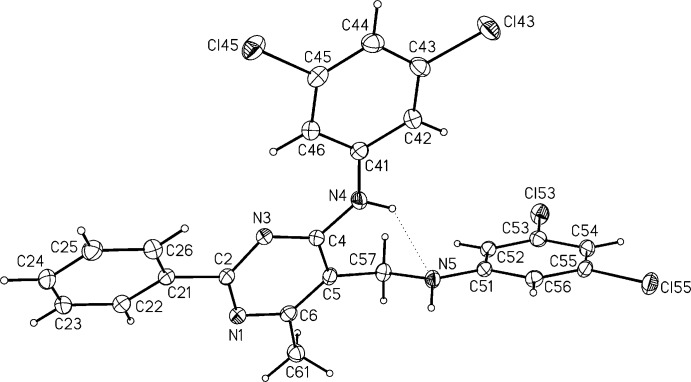
The molecule of the title compound, showing the atom-numbering scheme. Displacement ellipsoids are drawn at the 50% probability level and H atoms are shown as small spheres of arbitrary radii. The dotted line indicates the intramolecular N—H···N hydrogen bond.

**Fig. 2.**
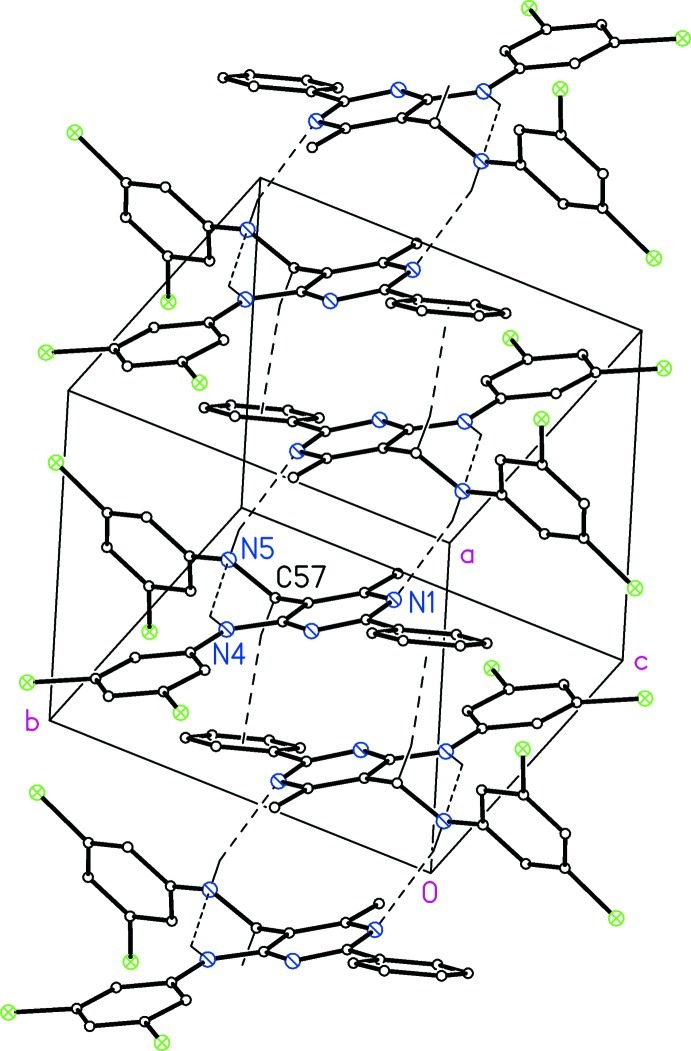
Part of the crystal structure of 5-[(3,5-dichloroanilino)methyl]-N-(3,5-dichlorophenyl)-6-methyl-2-phenylpyrimidin-4-amine, showing N—H···N and C—H···π interactions. Dashed lines indicate intra- and intermolecular hydrogen bonds. H atoms not involved in hydrogen bonding have been omitted for clarity.

## supplementary crystallographic information

### Comment

In this work, we extend the earlier analysis of 6-methyl-2-phenyl-5-substituted pyrimidine derivatives (Cieplik *et al.*, 2003, 2008, 2012) and report the crystal strucure of this class of derivatives, namely 5-[(3,5-dichloroanilino)methyl]-*N*-(3,5-dichlorophenyl)-6-methyl-2-phenylpyrimidin-4-amine.

The title compound crystallizes in *P*1 space group, with one molecule in the asymmetric unit (Fig. 1). There is an intramolecular N—H···N hydrogen bond between N4—H4 and N5 (Table 1), which closes six-memebered ring. This S(6) motif was observed in earlier described similar compounds (Cieplik *et al.*, 2006). The conformation of the title molecule is best defined by dihedral angles formed between the pyrimidine ring plane and the planes of the phenyl ring attached to the atom C2 and two other aryl rings of the (3,5-dichlorophenyl)amino or the (3,5-dichlorophenyl)aminomethyl groups attached, respectively, to the atoms C4 or C5 of the pyrimidine ring. These dihedral angles are 19.1 (2), 4.1 (2) and 67.5 (2)°, respectively.

The molecules are linked by a combination of N—H···N, C—H···π (Table 1) and also aromatic π–π stacking interactions. The N5 amine atom acts as hydrogen-bond donor to the pyrimidine atom N1 of adjacent molecule at (-*x* + 1, -*y* + 1, -*z* + 1). This result in the formation of a dimer *via* a cyclic *R*^2^_2_(12) ring motif. Additionally, the C57—H572 groups acts as a donor of C—H···π(arene) interaction to the benzene C21—C26 ring (-*x*, -*y* + 1, -*z* + 1). The combination of N—H···N and C—H···π interactions generates a chain running along the [100] direction (Fig. 2). Between C41—C46 rings of molecules of adjacent chains related by translation along a direction, there is also an aromatic π–π stacking interaction. The distance between the ring centroids of molecules at (*x*, *y*, *z*) and (-*x*, -*y* + 2, -*z* + 1) is 3.666 (4) Å with an interplanar spacing of 3.486 (4) Å and a centroid offset of 1.13 Å.

### Experimental

The title compound was obtained by adopting the procedure described previously by Cieplik *et al.* (2003). 4 g (0.010 mmol) of 4-(3,5-dichlorophenyl)amine-5-chloromethyl-2-phenyl-6-methylpyrimidine was dissolved in 50 ml of chloroform, and 2 g of 3,5-dichloroaniline. The reaction mixture was refluxed for 7 h with vigorous stirring, then was cooled and poured into 100 ml of water. The aqueous solution was extracted three times with chloroform (50 ml). The combined chloroform phases were dried over MgSO_4_, filtered and concentrated under vacuum. The oily residue was purified by column chromatography on silica gel (200–400 mesh) using CHCl_3_ as the eluent and by crystallization from methanol to give single crystals (yield: 4.37 g, 82.1%, m.p. 380–382 K).

### Refinement

The N-bonded H atoms were found from difference Fourier maps and refined with *U*_iso_(H) = 1.2*U*_eq_(N). The remaining H atoms were treated as riding on their carrier atoms, with C—H distances in the range 0.95–0.99 Å, and refined with *U*_iso_(H) = 1.2*U*_eq_(C), except methyl groups where *U*_iso_(H) = 1.5*U*_eq_(C).

### Crystal data

**Table:**

C_24_H_18_Cl_4_N_4_	*Z* = 2
*M**_r_* = 504.22	*F*(000) = 516
Triclinic, *P*1	*D*_x_ = 1.481 Mg m^−^^3^
Hall symbol: -P 1	Mo *K*α radiation, λ = 0.71073 Å
*a* = 8.267 (3) Å	Cell parameters from 12129 reflections
*b* = 11.271 (4) Å	θ = 4.3–32.6°
*c* = 12.788 (4) Å	µ = 0.54 mm^−^^1^
α = 76.53 (5)°	*T* = 85 K
β = 78.95 (5)°	Plate, yellow
γ = 81.42 (5)°	0.23 × 0.14 × 0.08 mm
*V* = 1130.4 (7) Å^3^	

### Data collection

**Table:**

Oxford Xcalibur PX with Onyx CCD diffractometer	5082 reflections with *I* > 2σ(*I*)
Radiation source: normal-focus sealed tube	*R*_int_ = 0.041
Graphite monochromator	θ_max_ = 32.6°, θ_min_ = 4.3°
φ and ω scans	*h* = −12→12
19215 measured reflections	*k* = −17→14
8041 independent reflections	*l* = −19→19

### Refinement

**Table:**

Refinement on *F*^2^	Primary atom site location: structure-invariant direct methods
Least-squares matrix: full	Secondary atom site location: difference Fourier map
*R*[*F*^2^ > 2σ(*F*^2^)] = 0.044	Hydrogen site location: inferred from neighbouring sites
*wR*(*F*^2^) = 0.100	H atoms treated by a mixture of independent and constrained refinement
*S* = 1.03	*w* = 1/[σ^2^(*F*_o_^2^) + (0.041*P*)^2^] where *P* = (*F*_o_^2^ + 2*F*_c_^2^)/3
8041 reflections	(Δ/σ)_max_ = 0.001
296 parameters	Δρ_max_ = 0.75 e Å^−^^3^
0 restraints	Δρ_min_ = −0.41 e Å^−^^3^

### Special details

**Table:**

Geometry. All e.s.d.'s (except the e.s.d. in the dihedral angle between two l.s. planes) are estimated using the full covariance matrix. The cell e.s.d.'s are taken into account individually in the estimation of e.s.d.'s in distances, angles and torsion angles; correlations between e.s.d.'s in cell parameters are only used when they are defined by crystal symmetry. An approximate (isotropic) treatment of cell e.s.d.'s is used for estimating e.s.d.'s involving l.s. planes.
Refinement. Refinement of *F*^2^ against ALL reflections. The weighted *R*-factor *wR* and goodness of fit *S* are based on *F*^2^, conventional *R*-factors *R* are based on *F*, with *F* set to zero for negative *F*^2^. The threshold expression of *F*^2^ > σ(*F*^2^) is used only for calculating *R*-factors(gt) *etc*. and is not relevant to the choice of reflections for refinement. *R*-factors based on *F*^2^ are statistically about twice as large as those based on *F*, and *R*- factors based on ALL data will be even larger.

### Fractional atomic coordinates and isotropic or equivalent isotropic displacement parameters (Å^2^)

**Table:**

	*x*	*y*	*z*	*U*_iso_*/*U*_eq_	
N1	0.26701 (17)	0.40318 (13)	0.58153 (11)	0.0170 (3)	
C2	0.1469 (2)	0.48670 (15)	0.61481 (13)	0.0155 (3)	
C21	0.0585 (2)	0.45458 (15)	0.72891 (13)	0.0168 (3)	
C22	0.0633 (2)	0.33165 (16)	0.78508 (14)	0.0198 (3)	
H22	0.1220	0.2687	0.7500	0.024*	
C23	−0.0180 (2)	0.30171 (17)	0.89231 (14)	0.0221 (4)	
H23	−0.0157	0.2183	0.9297	0.026*	
C24	−0.1019 (2)	0.39319 (18)	0.94450 (14)	0.0240 (4)	
H24	−0.1558	0.3725	1.0179	0.029*	
C25	−0.1074 (2)	0.51528 (17)	0.88948 (14)	0.0225 (4)	
H25	−0.1648	0.5779	0.9254	0.027*	
C26	−0.0289 (2)	0.54593 (16)	0.78179 (13)	0.0196 (3)	
H26	−0.0347	0.6293	0.7441	0.024*	
N3	0.09912 (17)	0.59756 (13)	0.55551 (11)	0.0165 (3)	
C4	0.1808 (2)	0.62576 (15)	0.45336 (13)	0.0163 (3)	
N4	0.14556 (18)	0.73910 (13)	0.38923 (12)	0.0194 (3)	
H4	0.215 (3)	0.7569 (17)	0.3309 (16)	0.023*	
C41	0.0217 (2)	0.83536 (15)	0.40395 (13)	0.0178 (3)	
C42	0.0187 (2)	0.93460 (15)	0.31393 (14)	0.0192 (3)	
H42	0.0956	0.9318	0.2488	0.023*	
Cl43	−0.09392 (6)	1.15946 (4)	0.20955 (4)	0.03017 (12)	
C43	−0.0962 (2)	1.03605 (16)	0.32040 (14)	0.0213 (4)	
C44	−0.2132 (2)	1.04310 (16)	0.41377 (15)	0.0223 (4)	
H44	−0.2922	1.1130	0.4176	0.027*	
Cl45	−0.35266 (6)	0.95034 (4)	0.61925 (4)	0.02676 (11)	
C45	−0.2088 (2)	0.94317 (16)	0.50077 (14)	0.0206 (4)	
C46	−0.0948 (2)	0.83949 (16)	0.49892 (14)	0.0200 (3)	
H46	−0.0956	0.7731	0.5604	0.024*	
C5	0.3055 (2)	0.54229 (15)	0.40746 (13)	0.0162 (3)	
C57	0.3832 (2)	0.57235 (15)	0.28867 (13)	0.0179 (3)	
H571	0.4663	0.5036	0.2719	0.021*	
H572	0.2965	0.5818	0.2427	0.021*	
N5	0.46367 (19)	0.68541 (14)	0.26224 (11)	0.0183 (3)	
H5	0.528 (3)	0.6832 (18)	0.3034 (16)	0.022*	
C51	0.5219 (2)	0.73560 (15)	0.15245 (13)	0.0164 (3)	
C52	0.4940 (2)	0.68769 (15)	0.06663 (13)	0.0175 (3)	
H52	0.4382	0.6164	0.0808	0.021*	
Cl53	0.50653 (6)	0.68927 (4)	−0.14584 (3)	0.02527 (10)	
C53	0.5497 (2)	0.74650 (16)	−0.03970 (13)	0.0184 (3)	
C54	0.6378 (2)	0.84794 (16)	−0.06569 (13)	0.0197 (3)	
H54	0.6773	0.8851	−0.1390	0.024*	
Cl55	0.77628 (5)	1.01867 (4)	−0.00557 (4)	0.02456 (10)	
C55	0.6651 (2)	0.89215 (15)	0.02165 (14)	0.0184 (3)	
C56	0.6071 (2)	0.84064 (15)	0.12903 (13)	0.0182 (3)	
H56	0.6244	0.8755	0.1863	0.022*	
C6	0.3436 (2)	0.43092 (15)	0.47665 (13)	0.0165 (3)	
C61	0.4707 (2)	0.33156 (16)	0.44054 (14)	0.0209 (3)	
H611	0.4296	0.2973	0.3881	0.031*	
H612	0.4904	0.2665	0.5039	0.031*	
H613	0.5745	0.3663	0.4061	0.031*	

### Atomic displacement parameters (Å^2^)

**Table:**

	*U*^11^	*U*^22^	*U*^33^	*U*^12^	*U*^13^	*U*^23^
N1	0.0158 (7)	0.0178 (7)	0.0174 (7)	−0.0026 (6)	−0.0028 (5)	−0.0029 (5)
C2	0.0135 (7)	0.0169 (8)	0.0161 (7)	−0.0038 (6)	−0.0020 (6)	−0.0024 (6)
C21	0.0139 (7)	0.0201 (8)	0.0159 (7)	−0.0034 (6)	−0.0027 (6)	−0.0016 (6)
C22	0.0166 (8)	0.0209 (9)	0.0209 (8)	−0.0029 (7)	−0.0032 (6)	−0.0017 (7)
C23	0.0180 (8)	0.0247 (9)	0.0207 (8)	−0.0058 (7)	−0.0045 (7)	0.0041 (7)
C24	0.0185 (8)	0.0361 (11)	0.0149 (8)	−0.0052 (8)	−0.0015 (6)	0.0000 (7)
C25	0.0184 (8)	0.0294 (10)	0.0189 (8)	−0.0003 (7)	−0.0014 (6)	−0.0063 (7)
C26	0.0197 (8)	0.0203 (9)	0.0183 (8)	−0.0021 (7)	−0.0029 (6)	−0.0031 (6)
N3	0.0139 (6)	0.0170 (7)	0.0172 (6)	−0.0017 (6)	−0.0023 (5)	−0.0012 (5)
C4	0.0147 (7)	0.0165 (8)	0.0170 (7)	−0.0032 (6)	−0.0036 (6)	−0.0002 (6)
N4	0.0181 (7)	0.0200 (7)	0.0157 (7)	0.0001 (6)	0.0008 (5)	0.0007 (6)
C41	0.0156 (8)	0.0184 (8)	0.0194 (8)	−0.0025 (7)	−0.0029 (6)	−0.0035 (6)
C42	0.0210 (8)	0.0173 (8)	0.0188 (8)	−0.0031 (7)	−0.0029 (6)	−0.0024 (6)
Cl43	0.0383 (3)	0.0190 (2)	0.0286 (2)	0.0018 (2)	−0.00697 (19)	0.00210 (17)
C43	0.0238 (9)	0.0152 (8)	0.0251 (9)	−0.0019 (7)	−0.0083 (7)	−0.0013 (7)
C44	0.0196 (8)	0.0182 (9)	0.0295 (9)	−0.0001 (7)	−0.0060 (7)	−0.0057 (7)
Cl45	0.0206 (2)	0.0292 (2)	0.0291 (2)	−0.00330 (18)	0.00381 (17)	−0.00958 (18)
C45	0.0165 (8)	0.0229 (9)	0.0230 (8)	−0.0040 (7)	−0.0004 (6)	−0.0073 (7)
C46	0.0183 (8)	0.0193 (8)	0.0209 (8)	−0.0034 (7)	−0.0027 (6)	−0.0009 (6)
C5	0.0147 (7)	0.0186 (8)	0.0148 (7)	−0.0053 (7)	−0.0002 (6)	−0.0024 (6)
C57	0.0184 (8)	0.0189 (8)	0.0160 (8)	−0.0042 (7)	−0.0005 (6)	−0.0037 (6)
N5	0.0184 (7)	0.0220 (7)	0.0141 (7)	−0.0063 (6)	−0.0025 (5)	−0.0005 (5)
C51	0.0131 (7)	0.0168 (8)	0.0163 (7)	0.0002 (6)	0.0007 (6)	−0.0012 (6)
C52	0.0154 (8)	0.0170 (8)	0.0190 (8)	−0.0021 (6)	−0.0013 (6)	−0.0028 (6)
Cl53	0.0286 (2)	0.0314 (2)	0.01672 (19)	−0.00648 (19)	−0.00104 (16)	−0.00705 (17)
C53	0.0177 (8)	0.0198 (8)	0.0170 (8)	0.0003 (7)	−0.0022 (6)	−0.0042 (6)
C54	0.0166 (8)	0.0220 (9)	0.0161 (8)	−0.0003 (7)	0.0017 (6)	0.0001 (6)
Cl55	0.0211 (2)	0.0214 (2)	0.0291 (2)	−0.00812 (17)	0.00107 (17)	−0.00176 (17)
C55	0.0131 (7)	0.0160 (8)	0.0238 (8)	−0.0027 (6)	0.0005 (6)	−0.0016 (6)
C56	0.0162 (8)	0.0181 (8)	0.0197 (8)	−0.0010 (7)	−0.0023 (6)	−0.0037 (6)
C6	0.0147 (7)	0.0185 (8)	0.0177 (8)	−0.0032 (6)	−0.0032 (6)	−0.0053 (6)
C61	0.0220 (9)	0.0211 (9)	0.0185 (8)	−0.0016 (7)	−0.0024 (7)	−0.0034 (7)

### Geometric parameters (Å, º)

**Table:**

N1—C2	1.341 (2)	C44—H44	0.9500
N1—C6	1.355 (2)	Cl45—C45	1.748 (2)
C2—N3	1.348 (2)	C45—C46	1.389 (3)
C2—C21	1.490 (2)	C46—H46	0.9500
C21—C26	1.398 (2)	C5—C6	1.389 (2)
C21—C22	1.403 (2)	C5—C57	1.512 (2)
C22—C23	1.394 (2)	C57—N5	1.465 (2)
C22—H22	0.9500	C57—H571	0.9900
C23—C24	1.386 (3)	C57—H572	0.9900
C23—H23	0.9500	N5—C51	1.405 (2)
C24—C25	1.390 (3)	N5—H5	0.81 (2)
C24—H24	0.9500	C51—C52	1.400 (2)
C25—C26	1.393 (2)	C51—C56	1.411 (2)
C25—H25	0.9500	C52—C53	1.390 (2)
C26—H26	0.9500	C52—H52	0.9500
N3—C4	1.340 (2)	Cl53—C53	1.7457 (18)
C4—N4	1.372 (2)	C53—C54	1.388 (2)
C4—C5	1.425 (2)	C54—C55	1.391 (2)
N4—C41	1.397 (2)	C54—H54	0.9500
N4—H4	0.85 (2)	Cl55—C55	1.7428 (19)
C41—C46	1.403 (2)	C55—C56	1.383 (2)
C41—C42	1.407 (2)	C56—H56	0.9500
C42—C43	1.381 (3)	C6—C61	1.508 (2)
C42—H42	0.9500	C61—H611	0.9800
Cl43—C43	1.739 (2)	C61—H612	0.9800
C43—C44	1.395 (3)	C61—H613	0.9800
C44—C45	1.388 (3)		
			
C2—N1—C6	116.77 (15)	C46—C45—Cl45	118.71 (14)
N1—C2—N3	126.46 (14)	C45—C46—C41	118.27 (16)
N1—C2—C21	116.89 (15)	C45—C46—H46	120.9
N3—C2—C21	116.64 (15)	C41—C46—H46	120.9
C26—C21—C22	119.13 (15)	C6—C5—C4	115.95 (14)
C26—C21—C2	120.75 (15)	C6—C5—C57	122.90 (15)
C22—C21—C2	120.12 (16)	C4—C5—C57	121.06 (15)
C23—C22—C21	120.12 (17)	N5—C57—C5	111.59 (14)
C23—C22—H22	119.9	N5—C57—H571	109.3
C21—C22—H22	119.9	C5—C57—H571	109.3
C24—C23—C22	120.26 (17)	N5—C57—H572	109.3
C24—C23—H23	119.9	C5—C57—H572	109.3
C22—C23—H23	119.9	H571—C57—H572	108.0
C23—C24—C25	120.02 (16)	C51—N5—C57	118.79 (14)
C23—C24—H24	120.0	C51—N5—H5	114.4 (14)
C25—C24—H24	120.0	C57—N5—H5	111.4 (14)
C24—C25—C26	120.14 (17)	C52—C51—N5	122.57 (15)
C24—C25—H25	119.9	C52—C51—C56	119.43 (15)
C26—C25—H25	119.9	N5—C51—C56	117.97 (15)
C25—C26—C21	120.32 (17)	C53—C52—C51	118.68 (15)
C25—C26—H26	119.8	C53—C52—H52	120.7
C21—C26—H26	119.8	C51—C52—H52	120.7
C4—N3—C2	116.02 (15)	C54—C53—C52	123.42 (16)
N3—C4—N4	119.79 (16)	C54—C53—Cl53	118.48 (13)
N3—C4—C5	122.54 (15)	C52—C53—Cl53	118.10 (13)
N4—C4—C5	117.68 (15)	C53—C54—C55	116.30 (15)
C4—N4—C41	132.19 (15)	C53—C54—H54	121.8
C4—N4—H4	114.8 (13)	C55—C54—H54	121.8
C41—N4—H4	112.9 (13)	C56—C55—C54	122.95 (16)
N4—C41—C46	125.49 (16)	C56—C55—Cl55	118.53 (14)
N4—C41—C42	115.01 (15)	C54—C55—Cl55	118.52 (13)
C46—C41—C42	119.50 (17)	C55—C56—C51	119.14 (16)
C43—C42—C41	119.91 (16)	C55—C56—H56	120.4
C43—C42—H42	120.0	C51—C56—H56	120.4
C41—C42—H42	120.0	N1—C6—C5	122.14 (16)
C42—C43—C44	121.94 (17)	N1—C6—C61	115.13 (15)
C42—C43—Cl43	119.13 (14)	C5—C6—C61	122.73 (15)
C44—C43—Cl43	118.93 (14)	C6—C61—H611	109.5
C45—C44—C43	116.89 (17)	C6—C61—H612	109.5
C45—C44—H44	121.6	H611—C61—H612	109.5
C43—C44—H44	121.6	C6—C61—H613	109.5
C44—C45—C46	123.47 (17)	H611—C61—H613	109.5
C44—C45—Cl45	117.81 (15)	H612—C61—H613	109.5
			
C6—N1—C2—N3	−2.8 (2)	Cl45—C45—C46—C41	−179.12 (13)
C6—N1—C2—C21	177.92 (14)	N4—C41—C46—C45	179.14 (16)
N1—C2—C21—C26	159.86 (15)	C42—C41—C46—C45	−0.6 (2)
N3—C2—C21—C26	−19.5 (2)	N3—C4—C5—C6	−2.8 (2)
N1—C2—C21—C22	−19.6 (2)	N4—C4—C5—C6	176.84 (15)
N3—C2—C21—C22	161.04 (15)	N3—C4—C5—C57	173.95 (15)
C26—C21—C22—C23	−0.2 (2)	N4—C4—C5—C57	−6.4 (2)
C2—C21—C22—C23	179.27 (15)	C6—C5—C57—N5	−124.24 (17)
C21—C22—C23—C24	−0.9 (3)	C4—C5—C57—N5	59.3 (2)
C22—C23—C24—C25	0.9 (3)	C5—C57—N5—C51	−171.30 (14)
C23—C24—C25—C26	0.1 (3)	C57—N5—C51—C52	5.4 (2)
C24—C25—C26—C21	−1.2 (3)	C57—N5—C51—C56	−176.58 (15)
C22—C21—C26—C25	1.2 (3)	N5—C51—C52—C53	177.13 (16)
C2—C21—C26—C25	−178.25 (16)	C56—C51—C52—C53	−0.8 (2)
N1—C2—N3—C4	0.1 (2)	C51—C52—C53—C54	2.7 (3)
C21—C2—N3—C4	179.38 (14)	C51—C52—C53—Cl53	−177.45 (13)
C2—N3—C4—N4	−176.81 (14)	C52—C53—C54—C55	−1.9 (3)
C2—N3—C4—C5	2.8 (2)	Cl53—C53—C54—C55	178.27 (13)
N3—C4—N4—C41	−8.2 (3)	C53—C54—C55—C56	−0.8 (3)
C5—C4—N4—C41	172.14 (17)	C53—C54—C55—Cl55	179.54 (13)
C4—N4—C41—C46	5.3 (3)	C54—C55—C56—C51	2.6 (3)
C4—N4—C41—C42	−174.89 (17)	Cl55—C55—C56—C51	−177.80 (13)
N4—C41—C42—C43	−178.50 (16)	C52—C51—C56—C55	−1.7 (2)
C46—C41—C42—C43	1.3 (2)	N5—C51—C56—C55	−179.73 (15)
C41—C42—C43—C44	−1.1 (3)	C2—N1—C6—C5	2.7 (2)
C41—C42—C43—Cl43	178.19 (13)	C2—N1—C6—C61	−176.51 (14)
C42—C43—C44—C45	0.2 (3)	C4—C5—C6—N1	−0.1 (2)
Cl43—C43—C44—C45	−179.07 (13)	C57—C5—C6—N1	−176.77 (15)
C43—C44—C45—C46	0.5 (3)	C4—C5—C6—C61	179.00 (15)
C43—C44—C45—Cl45	179.35 (13)	C57—C5—C6—C61	2.3 (2)
C44—C45—C46—C41	−0.3 (3)		

### Hydrogen-bond geometry (Å, º)

*Cg*1 is the centroid of the C21–C26 ring.

**Table:**

*D*—H···*A*	*D*—H	H···*A*	*D*···*A*	*D*—H···*A*
N4—H4···N5	0.85 (2)	2.19 (2)	2.875 (3)	137.6 (17)
N5—H5···N1^i^	0.81 (2)	2.40 (2)	3.171 (3)	158.1 (19)
C57—H572···*Cg*1^ii^	0.99	2.65	3.62 (2)	166

Symmetry codes: (i) −*x*+1, −*y*+1, −*z*+1; (ii) −*x*, −*y*+1, −*z*+1.
